# Changes in the fecal microbiome of the Yangtze finless porpoise during a short-term therapeutic treatment

**DOI:** 10.1515/biol-2020-0032

**Published:** 2020-06-05

**Authors:** Lei You, Congping Ying, Kai Liu, Xizhao Zhang, Danqing Lin, Denghua Yin, Jialu Zhang, Pao Xu

**Affiliations:** Wuxi Fisheries College, Nanjing Agricultural University, Wuxi, China; Scientific Observing and Experimental Station of Fishery Resources and Environment in the Lower Reaches of the Changjiang River, Ministry of Agriculture and Rural Affairs, Freshwater Fisheries Research Center, CAFS, Binhu District, Wuxi, Jiangsu, China

**Keywords:** Yangtze finless porpoise, fecal microbiome, therapeutic treatment, potential pathogens

## Abstract

The fecal microbiome is an integral part of aquatic mammals, like an inner organ. But we know very little about this inner organ of the threatened aquatic species, Yangtze finless porpoise (YFP). Four YFPs were placed into a purse seine for skin ulceration treatment, and this opportunity was taken to nurse the animals closer. In particular, we collected the feces of the YFPs before and after the paired healing and therapeutic treatment, along with samples of their fish diet and water habitat, to explore the changes in their fecal microbiome. Firmicutes (20.9–96.1%), Proteobacteria (3.8–78.7%), Actinobacteria (0.1–35.0%) and Tenericutes (0.8–17.1%) were the most dominant phyla present in the feces. The proportion of Proteobacteria and Actinobacteria increased after the treatment. Firmicutes showed a significant decrease, and most potential pathogens were absent, which reflected the administration of ciprofloxacin hydrochloride. Moreover, environmental shifts can also contribute to changes in the fecal microbiome. These results indicate that certain microbial interactions can be affected by environmental shifts, dietary changes and health-care treatments, which can also help maintain the internal environment of YFPs. These findings will inform the future enhanced protection and management of endangered YFPs and other vulnerable aquatic animals.

## 1 Introduction

The Yangtze finless porpoise (*Neophocaena asiaeorientalis* ssp. *asiaeorientalis*; YFP) is the only freshwater subspecies of *Neophocaena*, thus possessing an important biological and ecological niche as a research subject. Since the last century, the population of YFPs has fallen sharply. The species is now extremely endangered, and their distribution shows patchiness [1]. In order to save this flagship species, all levels of the state departments and scientific research institutions have actively been implementing rescue and protection measures. Since the release of five YFPs to Tian-E Zhou Baiji National Natural Reserve in 1990, four YFPs *ex situ* conservation populations have been established: one in the old channel of Shishou Tian-E Zhou, one in Tongling Tiebanzhou, one in the old channel of Jianli Hewangmiao and one at Anqing Xijiang. Today, and the total number of *ex situ* YFPs has exceeded 100; thus, this method has been crucial in YFP preservation, breeding, scientific research, etc. At Anqing Xijiang YFP *ex situ* conservation base, the YFP population was established in November 2016. Up to now, 18 YFPs inhabit the Xijiang River.

Dolphin fecal microbiomes have been shown to reflect their marine environment [2]. At the same time, fecal microbiomes are also affected by many factors. Some scholars have explored the gut microbiome of aquatic mammals such as baleen whale, sperm whale, seal and sea lion [3,4,5,6], but we still know very little about the key factors influencing fecal microbial communities in freshwater aquatic mammals, including YFPs. The classification and function of baleen whales’ fecal microbes are known to be influenced by diet and host species [5]. Similarly, the influential factors of fecal microbiome in sperm whales include dietary and host specificities [4]. Significant differences have been observed in the fecal microbiome of seals among different age groups [6]. Differences in host habitat [7], feeding habits and phylogeny lead to differences in the microbial composition of the distal gut in sea lions [3]. In sum, major determinants affecting the fecal microbiome of marine mammals include age, diet, host species and environment [8]. Additionally, some studies have reported that vitamins and antibiotics also affect the fecal microbiome. Antibiotics, such as ciprofloxacin [9], which are commonly used as human and animal medicine, can easily lead to changes in the fecal microbiome [10]. Vitamins can enhance existing fecal microbiome in mammals and effectively inhibit pathogens [11].

Notably, there have been few studies on the fecal microbiome of YFPs. The fecal microbiome structures of YFPs have been described according to their different living environments (Poyang Lake, Tian-E Zhou Baiji National Natural Reserve and Wuhan Baiji Dolphinarium) [12,13], which indicate that the fecal microbiome of YFPs is affected by their habitat. In contrast, in this study, 16S rRNA gene sequencing analysis was used to find the commonalities and differences in the composition of YFP fecal microbiome before and after a short-term therapeutic treatment, to identify possible influential factors and to explore the existence of potential pathogens.

## 2 Materials and methods

### 2.1 Background of animals

**Figure 1:**
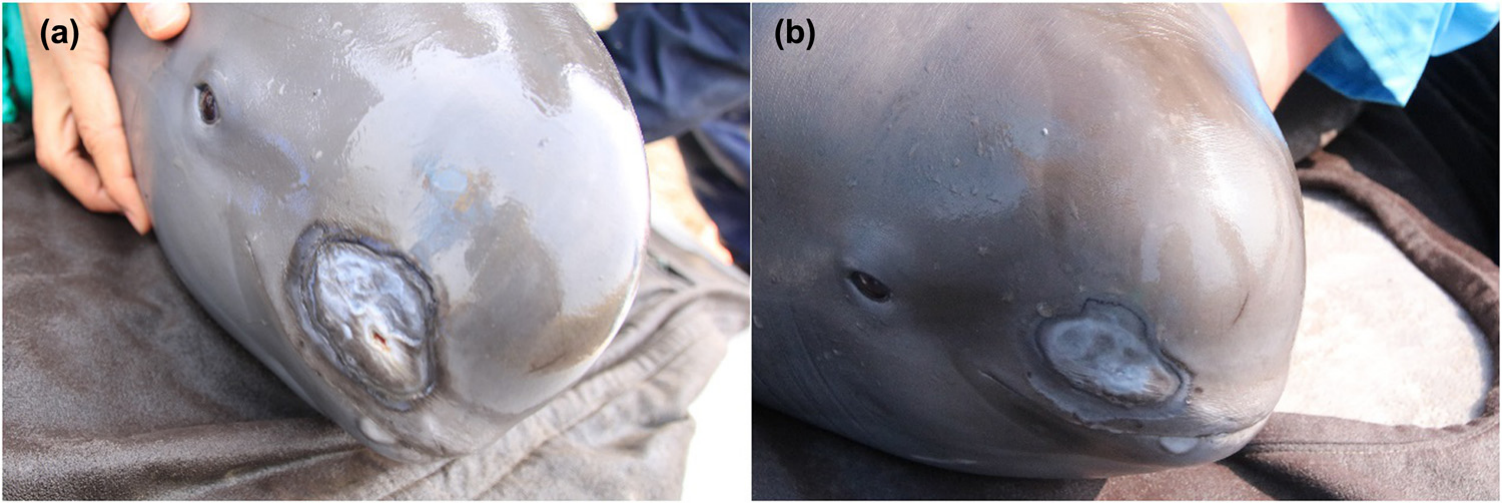
The typical appearance of YFP before and after treatment. (a) YFP before treatment and (b) YFP after treatment.

In December 2017, the Freshwater Fisheries Research Center began inspecting the population of YFPs in the Xijiang River. During the inspection, small areas of skin on both the head and tail fin of four YFPs were found to have ulcerations, scratches which were attributed to coastal beach rubble approached during predation (Figure 1). After slowly using net to surround four YFPs from outside water to near shore, the animals were safely held and lifted up from water by a specially made stretcher. To protect these injuries from infection by pathogens, a health examination, combining the on-site external application of drug therapy and a feeding regiment, was performed immediately. All four animals were male, and we named them A, B, C and D. According to the body length and the formula derived by Zhang [14], we estimated the age of the four animals afterward (Table 1). The age of YFP A, B, C and D was 10, 4, 13 and 5 years old, respectively.

**Table 1:** Animal description

Name	Sex	Overall length (cm)	Body length (cm)	Weight (kg)	Age (year)	Source
A	Male	169	160	48.5	10	Xijiang
B	Male	149	142	36.3	4	Xijiang
C	Male	176	166	57.1	13	Xijiang
D	Male	158	146	41.8	5	Xijiang

The whole procedure has two phases: on-site health examination and follow-up treatment. During the health examination, we measured animals’ body length and weight and also collected fecal samples from the intestine. Besides, a mixture of Chinese traditional herbs, Yunnan Baiyao (a famous Chinese patent medicine made of precious herbs, which can relieve blood stasis, promote blood circulation and ease pain and swelling [15]), and erythromycin ointment (the mixture of Yunnan Baiyao and erythromycin ointment in a ratio of 1 to 5) was applied to the wound site, and then drug coating was implemented for half an hour on a large suitable water-soaked sponge pad (Table 2). Then, the animals were released back to water in a purse seine (the water surface area of about 500 m^2^) temporarily for the follow-up treatment. The whole health examination was carried out by professional veterinarians from beginning to end, and the animals were unharmed and handled safely during the process.

**Table 2:** Therapeutic treatment information

Drug name	Time of day administered			Function	Source
	08:30 am	12:30 pm	17:30 pm		
Yunnan Baiyao	Health examination			External application, anti-inflammatory	Yunnan Baiyao Group Co, Ltd
Erythromycin Ointment	Health examination			External application, anti-inflammatory	Fuyuan Pharmaceutical Co, Ltd
Ciprofloxacin hydrochloride (mg)	4 × 0.25	—	4 × 0.25	Oral, health care	Baiyunshan Pharmaceutical Group Co, Ltd
Multivitamins (particle)	—	4	—	Oral, health care	Sino-US Shanghai Squibb Pharmaceutical Co, Ltd

The follow-up treatment commenced from 8th February to 5th March and lasted for 25 days. YFPs with abrasions were temporarily fed and observed by animal-care experts while living in a purse seine. Healthy and fresh crucians (bought from Anqing Yichuan Aquaculture Farm) were fed to the YFPs three times a day (8:30 am, 12:30 pm and 5:30 pm) after soaking in 5% salt water for 30 min to inhibit potential pathogens [16]. Each YFP was fed 1–2 kg every time. To prevent infection and promote wound healing, ciprofloxacin hydrochloride tablets (5 days) and multivitamin tablets (25 days) were placed in the mouth of bait fish to be fed to the YFPs on the appropriate days (Table 2).

After aforesaid temporary treatment, all animals were observed to swim normally and to eat steadily. We found that the ulceration of animals had improved remarkably and tended to heal. On the last day, after applying the mixed drugs and conducting the health examination one more time, the YFPs were released into open water.


**Ethical approval:** The research related to animal use has been complied with all the relevant national regulations and institutional policies for the care and use of animals. The medical examination and the relevant experiments performed in this study were approved by the Anqing Fisheries Bureau of China, the department responsible for the protected area. The research complies with the Aquatic Animal Protection Act promulgated in 1993.

### 2.2 Sample collection

We collected three kinds of samples during the whole process: feces (intestinal content), water and bait fish. The fecal samples and water samples were collected through the health examination before and after the treatment. The bait fish samples were collected during the follow-up treatment (Table 3).

**Table 3:** Sample information

Group	Sample ID	Type	Source	Date of sampling
FH group	A1	Intestinal content	YFP-A	02/08/2018
FH group	B1	Intestinal content	YFP-B	02/08/2018
FH group	C1	Intestinal content	YFP-C	02/08/2018
FH group	D1	Intestinal content	YFP-D	02/08/2018
FT group	A2	Intestinal content	YFP-A	03/05/2018
FT group	B2	Intestinal content	YFP-B	03/05/2018
FT group	C2	Intestinal content	YFP-C	03/05/2018
FT group	D2	Intestinal content	YFP-D	03/05/2018
F group	F1	Fish	CA-1	02/09/2018
F group	F2	Fish	CA-2	02/22/2018
F group	F3	Fish	CA-3	03/04/2018
W group	W1	Water	Water-1	02/08/2018
W group	W2	Water	Water-2	03/05/2018

First, during the non-harmful, non-invasive fecal collection, each YFP was kept on a large suitable water-soaked sponge pad and held in a steady position. The anus was cleaned with 70% ethanol. All instruments used in the experiment were sterilized with 75% alcohol and UV treatment, which is also illustrated in the method. Before inserting, we lubricated the head of the hose and cleaned the anal attachment with a sterile cotton swab. Then, the tube was inserted about 20 cm into the anus. The tube was then removed with the feces and cut into sections. The sections were put in a sterilized 2 mL Eppendorf tube and stored at −198°C (liquid nitrogen) until DNA extraction. There were eight fecal samples in total, which were divided into two groups; four representing the free-hunting (FH) group (A1, B1, C1 and D1) collected before the treatment and four representing the feeding treatment (FT) group (A2, B2, C2 and D2) collected after the treatment (Table 3).

Then, we collected one water sample at each health examination individually (W1 and W2): we took 150 mL of water inside purse seine into a sterile bottle and then passed the water through a 0.22 µm filter [17]. The filters were stored in 100% alcohol and were frozen at below −20°C until DNA extraction.

We also collected three bait fish samples during the follow-up treatment (F1, F2 and F3): bait fish was sampled by homogenizing the representative daily animal ration in a commercial food-grade blender, then a subsample of the homogenate was taken [18]. The homogenate was put in a sterilized 2 mL Eppendorf tube and stored at −198°C (liquid nitrogen) until DNA extraction.

### 2.3 DNA extraction

Total microbial genomic DNA samples were extracted using the DNeasy PowerSoil Kit (QIAGEN, Inc, Netherlands), following the manufacturer’s instructions and stored at −20°C for further analysis. The quantity and quality of extracted DNAs were measured using a NanoDrop ND-1000 spectrophotometer (Thermo Fisher Scientific, Waltham, MA, USA) and agarose gel electrophoresis, respectively.

### 2.4 16S rRNA Gene amplicon sequencing

PCR amplification of the bacterial 16S rRNA genes V3–V4 region was performed using universal primers (341F [ACTCCTACGGGAGGCAGCAG] and 806R [GGACTACHVGGGTWTCTAAT]) [19]. Sample-specific 7 bp barcodes were incorporated into the primers for multiplex sequencing. The PCR components contained 5 μL of Q5 reaction buffer (×5), 5 μL of Q5 High-Fidelity GC buffer (×5), 0.25 μL of Q5 High-Fidelity DNA Polymerase (5 U/μL), 2 μL (2.5 mM) of dNTPs, 1 μl (10 µM) of each forward and reverse primer, 2 μL of DNA template and 8.75 μL of ddH_2_O [20]. Thermal cycling consisted of initial denaturation at 98°C for 2 min, followed by 25 cycles consisting of denaturation at 98°C for 15 s, annealing at 55°C for 30 s and extension at 72°C for 30 s, with a final extension for 5 min at 72°C. PCR amplicons were purified with Agencourt AMPure Beads (Beckman Coulter, Indianapolis, IN, USA) and quantified using the PicoGreen dsDNA Assay Kit (Invitrogen, Carlsbad, CA, USA). After the individual quantification step, amplicons were pooled in equal amounts, and paired-end 2 × 300 bp sequencing was performed using the Illlumina MiSeq platform with MiSeq Reagent Kit v3 at Shanghai Personal Biotechnology Co, Ltd (Shanghai, China). All sequences, generated by high-throughput sequencing, were submitted to NCBI Sequence Read Archive under the accession numbers SRR8517807–SRR8517819.

### 2.5 Sequence analysis

The Quantitative Insights Into Microbial Ecology (QIIME, v1.8.0) pipeline was employed to process the sequencing data, as previously described [21]. Briefly, raw sequencing reads with exact matches to the barcodes were assigned to respective samples and identified as valid sequences. The low-quality sequences were filtered using the following criteria [22,23]: sequences that had a length of <150 bp, sequences that had average Phred scores of <20, sequences that contained ambiguous bases and sequences that contained mononucleotide repeats of >8 bp. Paired-end reads were assembled using FLASH [24]. After chimera detection, the remaining high-quality sequences were clustered into operational taxonomic units (OTUs) at 97% sequence identity by UCLUST [25]. A representative sequence was selected from each OTU using default parameters. OTU taxonomic classification was conducted by BLAST searching the representative sequences set against the Greengenes Database [26] using the best hit [27]. An OTU table was further generated to record the abundance of each OTU in each sample and the taxonomy of these OTUs. OTUs containing <0.001% of total sequences across all samples were discarded. To minimize the difference of sequencing depth across samples, an averaged, rounded rarefied OTU table was generated by averaging 100 evenly resampled OTU subsets under the 90% of the minimum sequencing depth for further analysis.

The alpha-diversity (the Chao1 estimator, the ACE estimator, Shannon diversity index and the Simpson index) and beta-diversity (principal coordinate analysis [PCoA] based on UniFrac distance [28]) of the samples were calculated by analyzing the species abundance, richness and clustering of samples. Using the Mothur software with the Metastats statistical algorithm [29,30], a pairwise comparison test was performed on the sequence size difference between groups at the phylum and genus levels. The significance of differentiation in microbial structure among groups was assessed by analysis of similarities (ANOSIM) [31,32] using R package “vegan”. *P* < 0.05 was considered a statistically significant difference. The Circos species relationship analysis was performed using the OmicShare tools (http://www.omicshare.com/tools).

A custom pathogen database was constructed by Apprill et al. [33], which included bacteria that had been identified as both marine mammal pathogens and human bacterial pathogens as recognized by the American Biological Safety Association. By comparing the result with the database, we tried to find the existence of all potential pathogens within the fecal samples of YFPs. Each sequence of uncultured and unclassified bacterial species was compared against the National Center for Biotechnology Information (NCBI) nucleotide collection (non-redundant nucleotide database) using Blastn with default parameters (https://blast.ncbi.nlm.nih.gov/Blast.cgi) on 18 December 2018. Then, we downloaded the 16S-sequence data of the known pathogens’ standard strains from the GenBank® nucleic acid sequence database of NCBI. The evolutionary history of potential pathogen OTUs and downloaded sequences was inferred using the neighbor-joining method [34]. The evolutionary distances were computed using the Kimura 2-parameter method and evolutionary analyses were conducted in MEGA 6.0.6 [35,36].

## 3 Results

### 3.1 General analysis of sequences obtained by high-throughput sequencing

**Figure A1:**
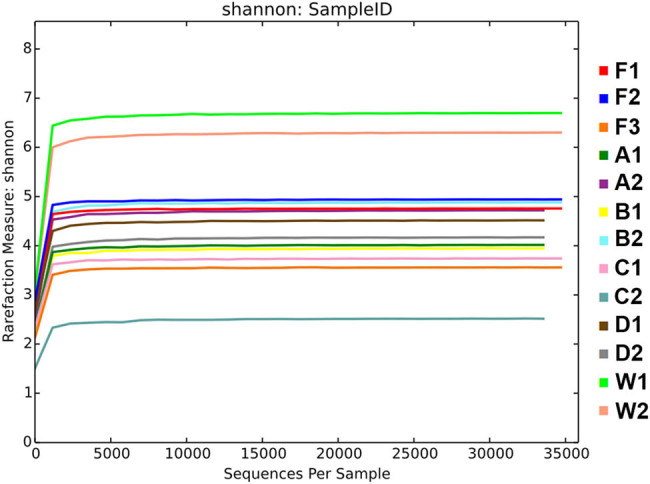
Rarefaction curves of intestinal contents, fish and water samples based on high throughput sequencing.

To explore the fecal bacteria structure and composition of the four YFPs during the short-term therapeutic treatment, 13 samples were analyzed with Illumina MiSeq. After removing the low-quality reads, a total of 1,148,664 effective high-quality reads were clustered into 1,998 OTUs within the 97% sequence similarity threshold. The number of OTUs at different annotated taxonomic levels is listed in Table 4. The stability of the rarefaction curves indicated that the sampling was reasonably representative, and the sequencing depth was adequate to cover the general bacterial diversity (Figure A1).

**Table 4:** Number of OTUs at different annotated taxonomic levels

Sample	Phylum	Class	Order	Family	Genus	Species	Unclassified
F1	241	240	240	218	158	31	0
F2	227	227	227	210	152	26	0
F3	248	248	248	229	173	43	0
A1	362	362	362	347	79	47	0
A2	540	540	539	518	215	98	0
B1	462	462	458	441	136	69	1
B2	473	473	468	443	128	37	1
C1	464	464	463	445	205	94	0
C2	365	365	365	358	162	30	0
D1	285	285	283	269	139	46	1
D2	441	441	439	423	131	89	0
W1	610	608	582	332	170	27	0
W2	588	587	564	317	150	19	0

Note: The numbers indicate the OTUs that were classified to specific taxonomy level.

### 3.2 Microbial community composition at the phylum level

**Figure 2:**
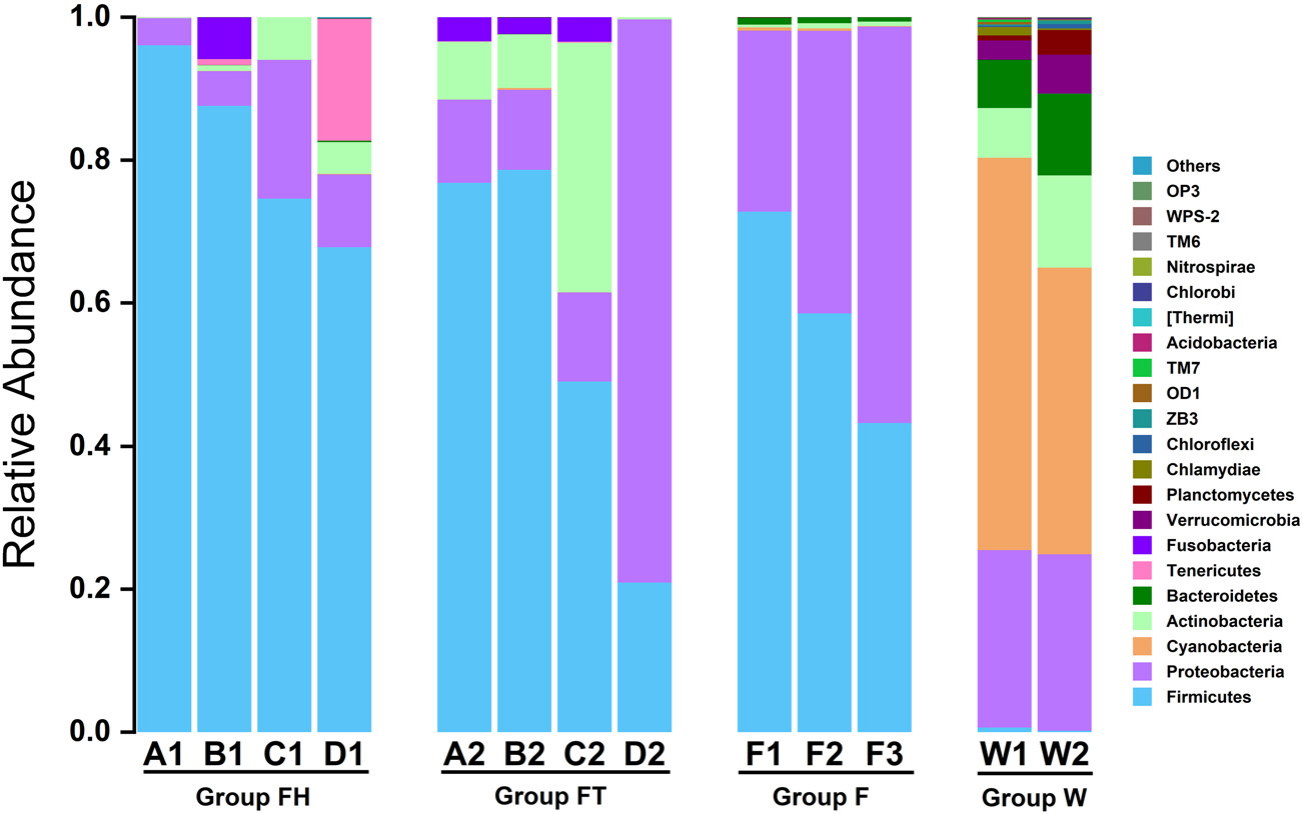
Relative abundance of bacterial phyla in fish, water and intestines. FH group represents feces of animals in the FH group, and FT represents feces of animals under human intervention. F represents fish samples, and W represents water samples.

The phylogenetic classification of sequences from all samples includes 22 different phyla or groups (Figure 2). In water samples, the dominant phyla were Cyanobacteria (varying from 40.0% to 54.8%), Proteobacteria (24.7–24.8%), Actinobacteria (6.9–12.9%), Bacteroidetes (6.8–11.5%), Verrucomicrobia (2.7–5.4%), Planctomycetes (0.7–3.5%) and Chlamydiae (0.2–1.2%), which represented 97.9–98.2% of the total reads. (The lower number in the bracket represents the least relative abundance in a single sample this phylum can account for, and the higher one indicates the most relative abundance in a single sample. The numbers referred to relative abundance in the bracket below represent the same.) In fish samples, the bacterial communities were dominated by Firmicutes (43.3–72.8%) and Proteobacteria (25.3–55.4%). These phyla were detected in high abundance and accounted for 98.1–98.7% of the total reads. The predominant phyla in the feces of YFPs were Firmicutes (20.9–96.1%), Proteobacteria (3.8–78.7%), Actinobacteria (0.1–35.0%), Fusobacteria (2.3–5.9%) and Tenericutes (0.8–17.1%), which covered 99.5–100.0% of the total reads.

**Figure 3:**
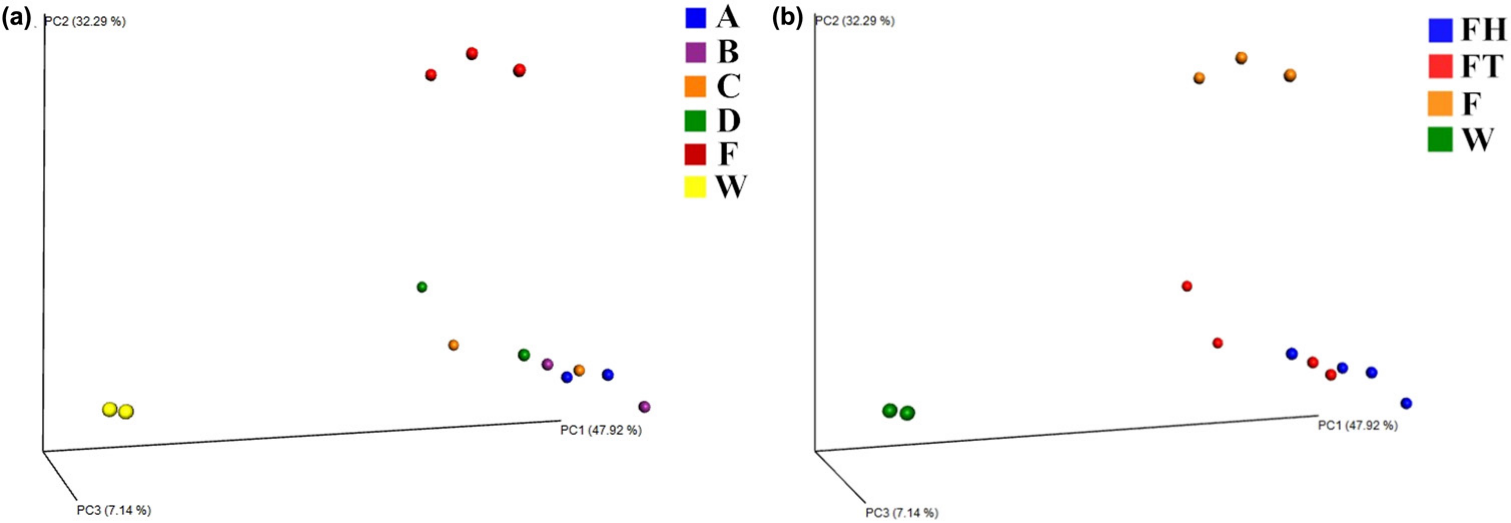
Weighted PCoA based on UniFrac distance in different groups. (a) Grouped by individual YFPs; (b) grouped by whether the animal obtained the therapeutic treatment. F represents fish samples and W for water samples.

A three-dimensional weighted PCoA based on UniFrac distance was obtained to measure the discrepancies and distances among all the samples (Figure 3). Bacterial samples displayed three clear clusters referring to fish, water and feces. With the PCoA, we observed the qualitative difference between each sample of feces. Each sample in the FT group deviates from the original (FH group) to a different degree (Figure 3a). In addition, from the FH group to FT group, we also detected that the tendency of the feces was associated with the fish and water samples (Figure 3b). A clear distinction in the bacterial community structure of different groups was also revealed by ANOSIM (*R* = 0.7923, *P* = 0.001).

### 3.3 Shared and unique bacteria in fecal microbiome reflect composition shifts during the therapeutic treatment

**Figure 4:**
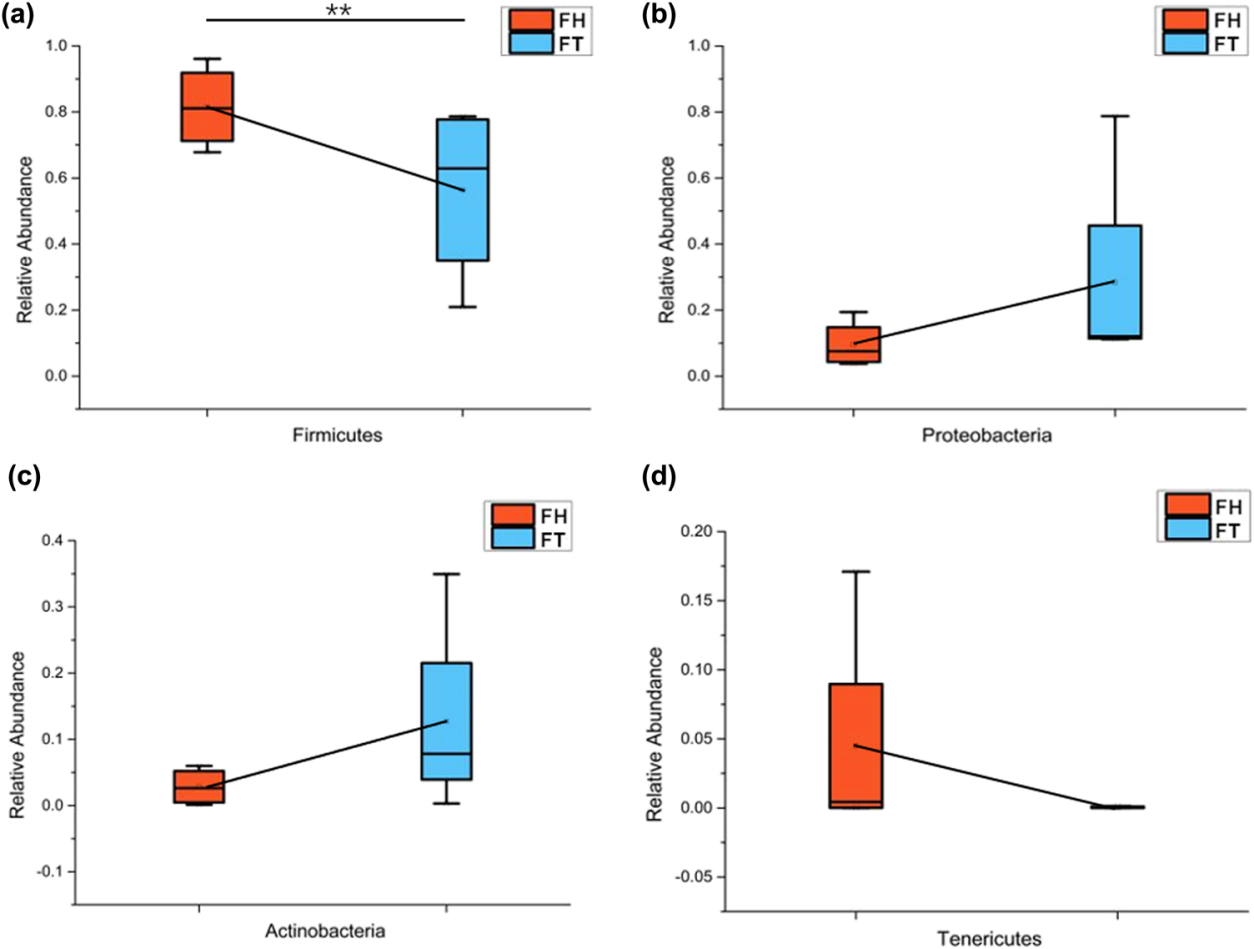
Changes in microbiome phyla of fecal samples throughout the therapeutic treatment: (a) Firmicutes, (b) Proteobacteria, (c) Actinobacteria and (d) Tenericutes.

**Figure 5:**
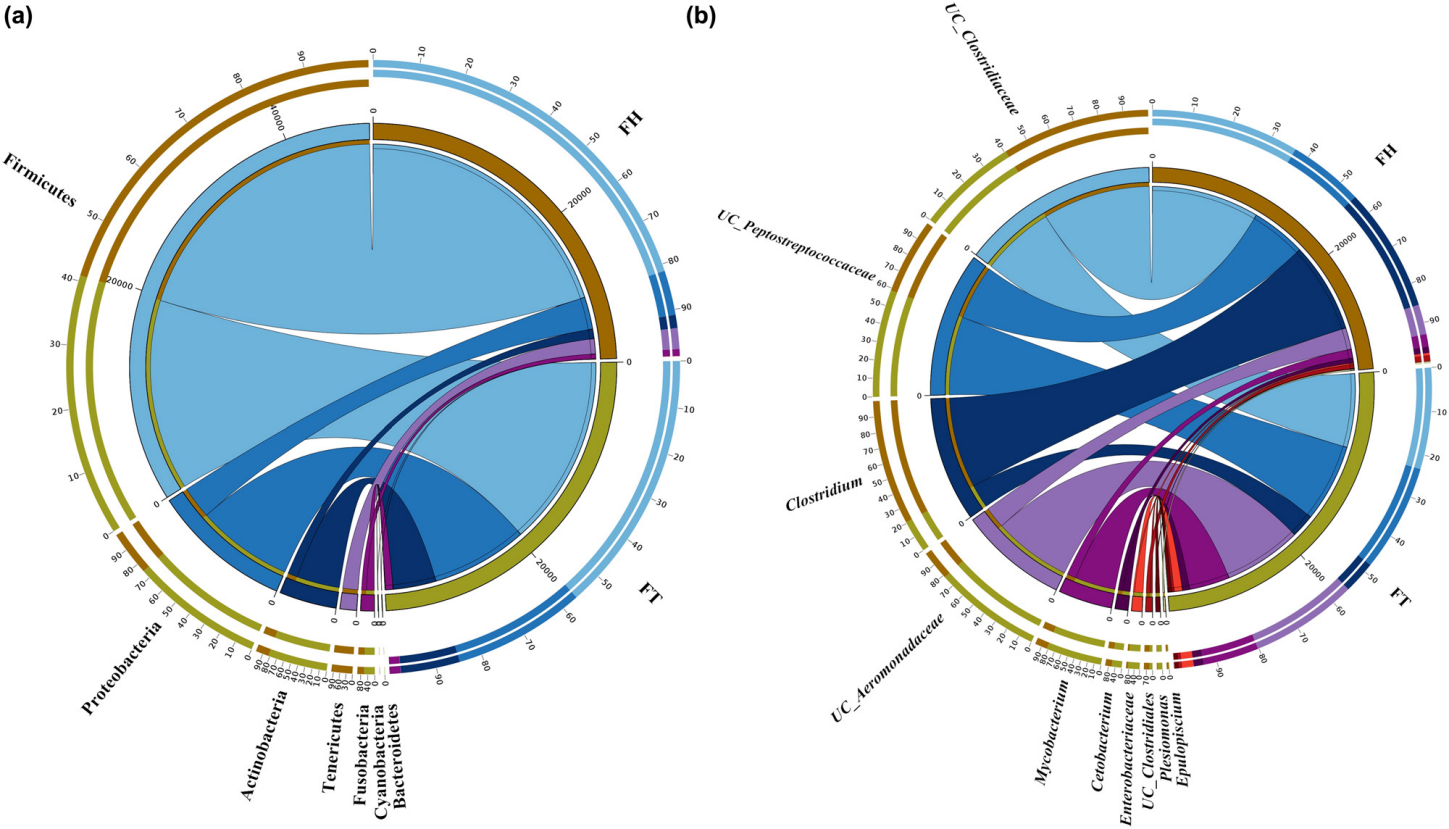
The Circos diagram of YFP sample groups: (a) phylum level and (b) genus level. UC represents unclassified. FH group represents feces of animals in the FH group, and FT represents feces of animals under human intervention.

Figure 4 shows that the fecal microbiome changes at the phylum level throughout the therapeutic treatment. The relative abundance of Firmicutes and Actinobacteria was higher in the FT group than in the FH group, whereas Proteobacteria and Tenericutes were relatively less abundant in the FT group. Among the two YFP groups, the relative abundance of Firmicutes differed significantly (Metastats, *P* = 0.031182). At the phylum level, both FH and FT groups were composed of seven shared phyla (Figure 5a). At the genus level, the top 10 shared bacterial genera in these two groups were Unclassified_*Clostridiaceae*, Unclassified_*Peptostreptococcaceae*, *Clostridium*, Unclassified_*Aeromonadaceae*, *Mycobacterium*, *Cetobacterium*, *Enterobacteriaceae*, Unclassified_*Clostridiales*, *Plesiomonas* and *Epulopiscium* (Figure 5b). In particular, *Mycobacterium* (Actinobacteria) and *Phormidium* (Proteobacteria) showed significant discrepancies, with *P*-values of 0.019026 and 0.036923, respectively (Table A1). Unique bacteria also existed in each group. For example, Bacteroidetes and Tenericutes only existed in the FH group, while Cyanobacteria only appeared in the FT group. At the genus level, unclassified_*Mycoplasmataceae*, *Edwardsiella*, *Acinetobacter*, *Sarcina* and unclassified_*Clostridia* only existed in the FH group, and *Erwinia* only appeared in the FT group.

In addition, the fecal microbiomes varied individually among the four YFPs. For instance, YFP-A and YFP-C shared similarities in microbial composition, as well as discrepancies. At the phylum level, their fecal microbiome showed an increase in Actinobacteria and a reduction in Firmicutes after the treatment. At the genus level, both YFP groups displayed an upward trend of *Mycobacterium* (Actinobacteria). However, Peptostreptococcaceae (Firmicutes) decreased from 37.94% to 7.05% in YFP-A after the treatment, and it rose significantly from 7.50% to 47.76% in YFP-C. Likewise, Aeromonadaceae (Firmicutes) was slightly augmented, 7.31%, in YFP-A and subtly reduced, 7.98%, in YFP-C after the treatment.

### 3.4 Identification of potential pathogens

**Figure 6:**
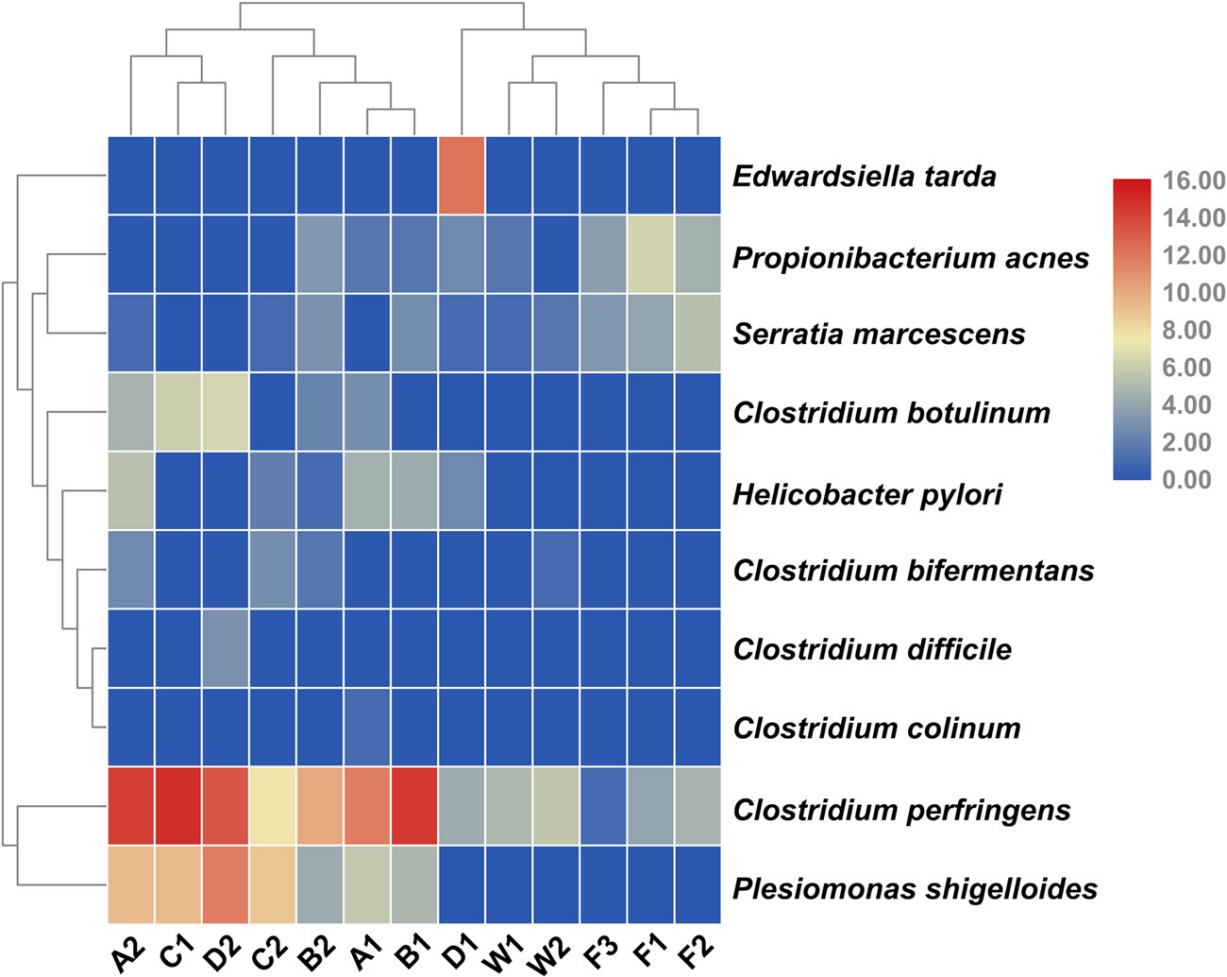
Heatmap analysis of potential pathogen genera of all samples. The color of the bar represents the abundance of each bacteria species in all samples. The longitudinal clustering indicates the similarity of all species among different samples.

**Figure A2:**
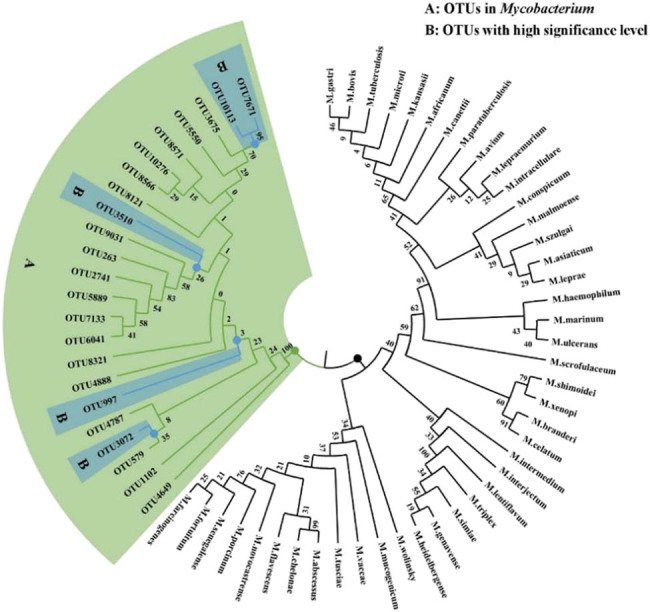
Neighbor-Joining tree showing sequences difference between OTUs of YFPs and pathogen species of *Mycobacterium*. Part A means that OTUs of YFPs in *Mycobacterium*; part B means OTUs with high significance level before and after the short-term feeding treatment; the remaining 43 branches are pathogens of *Mycobacterium* according to the data reported before.

To ensure the health of these YFPs during the short-term holding period, we identified the potential pathogens in all collected fecal samples. Through comparison with the pathogen database [33], 13 potential pathogen genera were screened out (*Mycobacterium*, *Propionibacterium*, *Bacillus*, *Streptococcus*, *Clostridium*, *Sphingomonas*, *Edwardsiella*, *Serratia*, *Acinetobacter*, *Pseudomonas*, *Stenotrophomonas*, *Helicobacter* and *Plesiomonas*). Then, we further screened the bacteria at species level through the pathogen database and found that there were many uncultured and unclassified species of *Mycobacterium*. The phylogenetic trees revealed that *Mycobacterium* species do not contain pathogenicity (Figure A2). Furthermore, we identified 10 potential pathogen species: *Clostridium bifermentans*, *Clostridium difficile*, *Clostridium botulinum*, *Clostridium colinum*, *Clostridium perfringens*, *Edwardsiella tarda*, *Helicobacter pylori*, *Plesiomonas shigelloides*, *Propionibacterium acnes* and *Serratia marcescens* (Figure 6). As it can be seen from Figure 6, both *C. perfringens* and *P. shigelloides* can be detected to various degrees in the total samples. *C. difficile* and *E. tarda* only appeared in YFP-D, and *E. tarda* disappeared after treatment. *C. colinum* only appeared in YFP-A. *H. pylori* was detected here in our fecal samples, and similarly, *Helicobacter* spp. and *Helicobacter cetorum* had been previously detected in YFP feces in an aquarium [37]. The potential pathogens detected in fish samples are as follows: *C. perfringens*, *P. acnes* and *S. marcescens.* The aforementioned potential pathogens were also detected in water samples, and *C. bifermentans* was also detected in W2 samples.

### 3.5 Microbial diversity increased during the therapeutic treatment

Multiple alpha diversity indices, including the Chao, ACE, Simpson and Shannon indices, were calculated to analyze the microbial community richness and evenness in the bacterial samples (Table 5 and Table A2). The fish and water samples were more diverse than the animal fecal samples. This indicates that the microbial diversity in the feces of the four YFPs was significantly higher after the treatment than before (paired-sample *t* test, *P* < 0.05).

**Table 5:** Alpha diversity indices of microbial communities in fish, water and intestines

Group	Sample ID	Simpson	Chao1	ACE	Shannon
FH	A1	0.93	227.00	227.00	4.76
	B1	0.85	374.78	388.29	4.02
	C1	0.94	253.00	254.03	4.94
	D1	0.73	241.00	241.00	3.56

FT	A2	0.87	571.53	580.53	4.72
	B2	0.84	300.80	316.01	3.74
	C2	0.84	505.01	522.42	3.95
	D2	0.90	395.44	390.97	4.88

F	F1	0.85	495.57	502.21	4.17
	F2	0.46	448.23	453.60	2.52
	F3	0.86	512.38	518.11	4.52

W	W1	0.96	619.69	619.62	6.30
	W2	0.98	597.67	599.37	6.70

## 4 Discussion

At present, the study of fecal microbes in aquatic mammals is relatively unusual [38]. The fecal microbiome plays an integral physiological role in host health, including the production of short-chain fatty acids, the synthesis of vitamins, the regulation of the immune system and the development of the brain [39,40,41,42], which is especially crucial for aquatic mammals like YFPs. Therefore, we took advantage of this short-term therapeutic treatment to explore the changes in the fecal microbiomes of YFPs and the most influential factors of intervention. We focused on the following three factors: water in the holding area, fish diet and health-care drugs including ciprofloxacin hydrochloride and multivitamins.

We found that the fecal microbiome diversity of YFP-B decreased after the treatment, while that of YFP-A, -C and -D showed an uptrend. During the treatment, the animals were treated with short-term ciprofloxacin hydrochloride for 5 days to ensure their safety and avoid wound infection. Generally speaking, the diversity of fecal microbiome of animals will be reduced due to the broad-spectrum bactericidal effect of ciprofloxacin hydrochloride during the treatment period, and the effective period of such antibiotics is about 7 days [43]. Different individuals have different responses to a drug, due to their different constitutions and adaptive adjustment abilities [44]. Similarly, the four YFPs showed different biases in their responses to the drug. Among them, YFP-B may still be affected due to its strong sensitivity to the drug and its inadequate self-adaptability, resulting in a downward trend in its fecal microbiome diversity. At the same time, for the other three YFPs, the diversity of fecal microbiome is on the rise. In addition to its own adaptive regulation ability to drugs, the difference in food intake may also be the cause of the discrepancy. If YFP-A, -C and -D ingested more bait fish than YFP-B during daily feeding, it would also increase the bacterial diversity (average 7.6 kg per day). In a word, after the treatment, the diversity of fecal microbial community of YFP-A, -C and -D increased after adaptive adjustment and stable exogenous intake. YFP-B may have a slight decrease in the diversity compared with that before treatment because of its strong sensitivity to the drugs and inadequate regulation.

The weighted PCoA principal coordinates of the bacterial compositions inside YFPs’ intestine, the water in the holding area and the fish diet were clearly classified into three clusters (Figure 3). This showed that the fecal microbiome of YFPs were relatively stable during the short-term treatment. However, the coordinates from the FT group were more skewed toward the F group than the data from the FH group, which proved that the fecal microbiome changed during the short-term therapeutic treatment. At the phylum level, we found that Firmicutes, Proteobacteria, Actinobacteria and Fusobacteria were the most dominant group of bacteria in the four YFPs. Compared with that in previous studies, Actinobacteria was also the dominant bacteria in feces from YFPs in Tian-E Zhou Baiji National Natural Reserve [12] and Wuhan Baiji Dolphinarium [13]. In contrast, the phylum Deinococcus–Thermus was found in the feces of 12 YFPs in Poyang lake, but was not detected in the individuals in this study [12]. The dominant phylum Bacteroidetes in Poyang lake YFPs has low relative abundance in our samples [12]. In addition, Fusobacteria was detected in our samples and the YFPs in the Dolphinarium [13], while it has not been detected in the intestines of wild YFPs (Poyang Lake [12]). However, whether this finding applies to YFPs in other unstudied habitats is yet to be known. Except for Firmicutes, the dominant fecal microbial bacteria, including Proteobacteria, Fusobacteria and especially Bacteroidetes, differed largely in other non-YFP marine carnivores [38]. For example, the fecal microbes of baleen whales have been shown to be mostly composed of the two phyla Bacteroidetes and Firmicutes [5], while the relative abundance of Bacteroidetes in each YFP sample ranged from 0.05% to nearly 0% in this study. Bacteroidetes is a phylum of Gram-negative bacteria that is found in many different niches [45] and helps maintain a healthy intestinal homeostasis. Previous studies have shown that a high salinity of inhabited water significantly augments the relative abundance of Bacteroidetes [46]. Therefore, unlike baleen whales or other marine carnivores, living in a highly saline ocean environment, it is likely that freshwater habitation may be responsible for the lower content of Bacteroidetes in the intestine of YFPs. These similarities and differences can be a perspective for further study.

During this short-term treatment, the fecal microbiome of the YFPs underwent significant changes within a stable condition. In some cases, the relative abundance of some fecal microbes increased significantly after the manual intervention. For example, the relative abundances of the three phyla, Proteobacteria, Actinobacteria and Fusobacteria, increased after the treatment. We found a large amount of Proteobacteria and a small amount of Actinobacteria among the fish for consumption, which may have led to an increase in the content of the two bacterial phyla in the YFP fecal microbiome after the treatment. In our study, the Fusobacteria is mainly composed of *Cetobacterium somerae* and unclassified_*Cetobacterium*. *C. somerae* is commonly found in the guts of freshwater fish and is capable of producing vitamin B12 [47]. We speculated that the increase in Fusobacteria may help the YFPs satisfy their need for vitamin B12. At the genus level, the relative abundance of *Mycobacterium* significantly increased from 2.8% to 12.7% after the therapeutic treatment. Due to the presence of 43 potential pathogens in *Mycobacterium*, we performed a phylogenetic tree analysis of the top 20 OTUs, the 5 significant differences in OTUs and 43 reported potential pathogens (Figure A2). The results show that the OTUs belonging to the YFPs were not on the same branch as the 43 potential pathogens, indicating that none of the *Mycobacterium* OTUs detected in the YFPs were a previously known potential pathogen, so they may not have even been pathogenic or they may have been less pathogenic. In contrast, the relative abundance of certain fecal microbiome declined. At the phylum level, the relative abundance of Firmicutes decreased significantly from 81.5% to 56.3% after the treatment. This may be related to the 5 days, when ciprofloxacin hydrochloride was administered to the YFPs. Studies have shown that ciprofloxacin hydrochloride can reduce the relative abundance of Firmicutes [48]. Ciprofloxacin hydrochloride also has strong permeability and is not susceptible to drug resistance [49]. It has a broad spectrum of antibacterial activity and has a bactericidal effect on Gram-positive and Gram-negative bacteria, including most pathogens [50]. Therefore, its presence also explains the reduction and cancellation in OTU numbers of potential pathogens, *C. colinum*, *E. tarda* and *P. acnes*. The dose of ciprofloxacin was small, and its lethality is not as strong as penicillin [51]. However, it can be seen from the results that it inhibits most potential pathogens (Figure 6). It can also be seen that *C. perfringens* had many OTUs across all samples, even being detected in the water samples. *C. perfringens* prefers to live in the intestines of many warm-blooded animals, including humans, and in warm soils. It can cause common foodborne illnesses and necrotic enteritis through food transmission [52]. Fortunately, its average relative abundance was significantly reduced in the YFPs after the therapeutic treatment (*C. perfringens*, mean [FH group] = 14706.5, mean [FT group] = 7725.7).

We also found that *H. pylori*, *C. bifermentans*, *C. difficile* and *S. marcescens* were detected in the feces of YFPs across two separate physical examinations, with their OTU counts almost unchanged. Among them, *H. pylori* is the cause of a variety of gastrointestinal diseases [53]. *Helicobacter* spp. was detected in the fecal samples of YFP at the Wuhan Baiji Dolphinarium, after the YFPs were fed with infected catfish [37]. However, *H. pylori* was not detected in the fish diet and water samples in our study, so we speculate that it might have originated in the wild habitat before arriving at the Anqing Xijiang YFP *ex situ* conservation base. *C. bifermentans* can produce both nutrients and toxins in the human body [54]. *C. difficile* contributes to the normal microbial community in some healthy individuals, but patients with infectious diarrhea are often susceptible to its pathogenic potential [55]. *S. marcescens* has also been detected in the gastrointestinal or upper respiratory tracts of healthy people [56]. In general, the aforementioned potential pathogens may pose a greater threat to immune-compromised individuals, but they were maintained at a very low level (0–0.08‰) in our four YFPs, and they displayed no pathological effect on their bodies under these circumstances. Therefore, the fecal microbiome of YFPs showed that the animals have the stability to be able to resist the low-abundance of potential pathogens. However, we need to stay alert and prevent the relative abundance of potential pathogens from growing over to a certain extent, thereby affecting the animals’ health [57]. Thus, this result has certain guidance to the work of Anqing Xijiang YFP *ex situ* conservation base.

The changes in the fecal microorganisms of the YFP also reflected the effects of environmental shifts. The 11 phyla, Firmicutes, Proteobacteria, Cyanobacteria, Actinobacteria, Bacteroidetes, Fusobacteria, Verrucomicrobia, Planctomycetes, Chlamydiae, [Deinococcus–Thermus] and Nitrospirae, in the fecal microbial community of YFP are shared with the water environment, which indicates that the fecal microbiome has a certain connection with the water environment. The living environment was shifted from open water area (Xijiang River, 9 km long) to semi-open water area (the water surface area near shore of about 500 m^2^). The background of the water environment of the animals did not change, but their range of activity became relatively fixed, so the external interference was minimal. Diet is one of the important factors affecting the fecal microbiome [5]. After the treatment, environmental changes also led to changes in feeding habits: from free predation to artificial feeding. Compared with free predation, artificial feeding makes the feeding sources of animals more stable and more conducive to the stable colonization of fecal microorganisms.

In conclusion, this study shows that the fecal microbiome of the YFPs is affected by environmental change, dietary change and health care during the short-term therapeutic treatment, which reveals that a crucial first step has been taken to understand the microbial communities in the intestine of YFPs and aid to benefit the host development and health. Future studies on the drivers of these common or host-specific fecal microbiomes and their physiological effects will provide further insight into the development and function of the YFP microbiome. This in-depth study of the fecal microbiome of the YFPs establishes an understanding of the healthy relationship between fecal microbes and YFPs, which provides scientific support for the further strengthening of YFP *ex situ* conservation work and improving the effectiveness of the protective measures.

## Appendix

**Table A1:** Significance of differences between FH and FT group microbiomes at the phylum and genus levels.

Taxonomy		Mean (FH)	Variance (FH)	Stderr (FH)	Mean (FT)	Variance (FT)	Stderr (FT)	*p*-value	*q*-value
Phylum	Firmicutes	0.81523	0.016162	0.063565	0.563558	0.074147	0.13615	0.031182	1
Genus	*Mycobacterium*	0.067106	0.003525	0.029687	0.432733	0.141326	0.187967	0.019026	1
	*Phormidium*	0.000106	0	0.000064	0	0	0	0.036923	1

**Table A2:** Alpha diversity indices of microbial communities in fish, water and fecal groups.

Group	Simpson	Chao1	ACE	Shannon
FH	0.86 ± 0.1	273.95 ± 68.06	277.58 ± 74.63	4.32 ± 0.64
FT	0.86 ± 0.03	443.2 ± 19.51	452.48 ± 120.69	4.32 ± 0.56
F	0.72 ± 0.23	485.39 ± 33.26	491.31 ± 33.61	3.74 ± 1.07
W	0.97 ± 0.01	608.68 ± 15.57	609.5 ± 14.32	6.5 ± 0.28
